# An Uncoupling of Canonical Phenotypic Markers and Functional Potency of *Ex Vivo*-Expanded Natural Killer Cells

**DOI:** 10.3389/fimmu.2018.00150

**Published:** 2018-02-02

**Authors:** Nicole A. P. Lieberman, Kole DeGolier, Kristen Haberthur, Harrison Chinn, Kara W. Moyes, Myriam N. Bouchlaka, Kirsti L. Walker, Christian M. Capitini, Courtney A. Crane

**Affiliations:** ^1^Ben Towne Center for Childhood Cancer Research, Seattle Children’s Research Institute, Seattle, WA, United States; ^2^Department of Pediatrics, Carbone Comprehensive Cancer Center, University of Wisconsin School of Medicine and Public Health, Madison, WI, United States; ^3^Department of Neurological Surgery, University of Washington, Seattle, WA, United States

**Keywords:** natural killer cells, immunotherapy, *ex vivo* expansion, adoptive transfer, clinical product, phenotypic analysis, functional analysis

## Abstract

Recent advances in cellular therapies for patients with cancer, including checkpoint blockade and *ex vivo*-expanded, tumor-specific T cells, have demonstrated that targeting the immune system is a powerful approach to the elimination of tumor cells. Clinical efforts have also demonstrated limitations, however, including the potential for tumor cell antigenic drift and neoantigen formation, which promote tumor escape and recurrence, as well as rapid onset of T cell exhaustion *in vivo*. These findings suggest that antigen unrestricted cells, such as natural killer (NK) cells, may be beneficial for use as an alternative to or in combination with T cell based approaches. Although highly effective in lysing transformed cells, to date, few clinical trials have demonstrated antitumor function or persistence of transferred NK cells. Several recent studies describe methods to expand NK cells for adoptive transfer, although the effects of *ex vivo* expansion are not fully understood. We therefore explored the impact of a clinically validated 12-day expansion protocol using a K562 cell line expressing membrane-bound IL-15 and 4-1BB ligand with high-dose soluble IL-2 on the phenotype and functions of NK cells from healthy donors. Following expansions using this protocol, we found expression of surface proteins that implicate preferential expansion of NK cells that are not fully mature, as is typically associated with highly cytotoxic NK cell subsets. Despite increased expression of markers associated with functional exhaustion in T cells, we found that *ex vivo*-expanded NK cells retained cytokine production capacity and had enhanced tumor cell cytotoxicity. The preferential expansion of an NK cell subset that is phenotypically immature and functionally pleiotropic suggests that adoptively transferred cells may persist better *in vivo* when compared with previous methods using this approach. *Ex vivo* expansion does not quell killer immunoglobulin-like receptor diversity, allowing responsiveness to various factors *in vivo* that may influence activation and inhibition. Collectively, our data suggest that in addition to robust NK cell expansion that has been described using this method, expanded NK cells may represent an ideal cell therapy that is longer lived, highly potent, and responsive to an array of activating and inhibitory signals.

## Introduction

Natural killer (NK) cells are cytotoxic effector lymphocytes of the innate immune system that are essential for the elimination of various pathogens and transformed cells (1). The role of NK cells in surveillance of transformed cells is supported by observations of an increased risk of cancer in patients with poor NK cell cytotoxic activity (2, 3), and murine studies have directly demonstrated NK cell-mediated tumor elimination (4–8). NK cells may be activated in response to stress-induced ligands, antibodies, or other activating proteins expressed on the surface of target cells, resulting in cytokine production, proliferation, and the release of cytolytic granules containing perforin and granzyme (9).

The interest in using NK cells as a cellular immunotherapy has led to an array of expansion protocols using long-term culture with recombinant cytokines or agonistic antibodies (10). Extended exposure of NK cells to soluble IL-15/IL-15Rα complexes increases in mature murine NK cells exhibiting replicative senescence and diminished cytolytic capabilities after 2 weeks (11). Protocols developed more recently have relied on feeder cell lines in addition to cytokines. A commonly used NK cell expansion clinical protocol uses irradiated K562 cells engineered to express membrane-bound IL-15 and membrane-bound 4-1BBL (K562-mb15-4-1BBL) (12). Studies using these cells demonstrate extensive NK cell expansion, increased activating receptor expression, and pro-inflammatory cytokine production (13, 14). It is not clear, however, whether NK cell subsets are equivalently expanded *ex vivo*, and the relative representation and contributions to antitumor immunity upon administration to patients.

Advances in cellular immunotherapy for patients with cancer using *ex vivo*-expanded T cells have highlighted the importance of their *in vivo* persistence to effectively control disease. NK cell approaches are faced with similar concerns, in particular because they do not generally undergo homeostatic proliferation *in vivo* (15). In addition, the percentages of NK cells that differentiate to memory cells, and the duration of their persistence, are diminished as compared with their T cell counterparts (16, 17). Further, the conditions required to achieve long-term memory in NK cells may not be recapitulated in patients with cancer, raising concerns of long-term persistence following *ex vivo* expansion and adoptive transfer.

Prolonged NK cell stimulation can occur as transformed cells accumulate (18, 19) or during chronic viral infections (20). Although the mechanisms for phenotypic and functional changes in NK cells following chronic stimulation are not fully defined, previous work demonstrates internalization of activating receptors following chronic stimulation (21), uncoupling of signaling adaptor proteins from activating surface receptors (22), and the downregulation of the transcription factor Eomesodermin (Eomes) in NK cells that can no longer control B cell lymphoma tumor growth (23). Consistent with these findings, patients with melanoma have decreased Eomes expression (24), suggesting that this may be a hallmark of NK cells with impaired pro-inflammatory functions. Using chronic stimulation to expand NK cells *ex vivo* may therefore result in a functionally impaired NK cell population, requiring greater numbers of NK cells to achieve efficacy, or subsequent selection of expanded NK cells to improve *in vivo* activation.

Given their demonstrated cytolytic capacity, we hypothesized that K562-mb15-4-1BBL *ex vivo*-expanded NK cells were mature, terminally differentiated, and may therefore have limited capacity for persistence, licensing, and responsiveness to the tumor microenvironment. Surprisingly, however, we find robust proliferation of a canonically less mature NK cell population that express several markers associated with less differentiated NK cells. This population also expresses several genes that enhance NK cells’ potential to differentiate and persist *in vivo*, including increased Eomes, granzymes A and K, and decreased granzyme B (GzmB). Together, our data suggest that *ex vivo*-expanded NK cells may have greater longevity *in vivo* than predicted based on both enhanced cytotoxicity following expansion and phenotypic and functional analysis of adoptively transferred T cells.

## Materials and Methods

All protocols have been reviewed and approved by the relevant institutional committees, including the Seattle Children’s Research Institute Institutional Biosafety Committee (Approval #1211) and Institutional Review Board (Approval #14412).

### Cell Lines, Cell Culture, and Peripheral Blood Mononuclear Cells (PBMCs)

K562 (human erythroblastoid cell line; American Type Culture Collection) and K562-mb15-4-1BBL (12) (a generous gift from Dr. Dario Campana and Dr. Lewis Lanier) were cultured in RPMI-1640 (ThermoFisher, Waltham, MA, USA) supplemented with 10% fetal bovine serum (FBS) (HyClone, Little Chalfont, UK) at 37°C in 5% CO_2_. Human PBMCs were isolated from healthy donors by centrifugation over a Ficoll gradient per the manufacturer’s instruction (STEMCELL Technologies, Vancouver, BC, Canada). PBMCs were stored long term in FBS + 10% DMSO and submerged in liquid nitrogen.

### Expansion of NK Cell Products

Quick-thawed (37°C) bulk PBMC were cultured at a 1:1 ratio with 100 Gy irradiated K562-mb15-4-1BBL in NK cell media containing X-VIVO-10 (Lonza, Basel, Switzerland) supplemented with 10% human AB serum (Corning Cellgro, Inc., Corning, NY, USA) and 1,000 U/mL recombinant human IL-2 (R&D Systems, Minneapolis, MN, USA) and incubated at 37°C and 5% CO_2_ on day 0. Cultures were expanded in T75 flasks with 10 mL media and IL-2, supplemented with fresh 10 mL media and cytokine on days 4, 7, and 10. All cells in culture were harvested on day 12 for subsequent phenotyping and functional assays. NK cells were donor matched for all measurements before and following expansion.

### NK Cell Isolation

Following thaw of PBMCs (day 0 NK cells) or after 12 days of coculture of bulk PBMCs with K562-mb15-4-1BBL, NK cells were isolated *via* CD14+ cell depletion with the use of a magnetic bead selection kit (STEMCELL Technologies), followed by NK cell enrichment using a magnetic bead negative selection kit (human NK cell isolation kit, Miltenyi Biotec, Bergisch Gladbach, Germany) according to the manufacturer’s protocol. Before functional studies, NK cells were rested in NK media for 24 h.

### Phenotypic Characterization by Flow Cytometry

Peripheral blood mononuclear cells or expanded NK cells were surface stained with panels of monoclonal antibodies used at the manufacturer’s recommended concentration. Antibodies from BioLegend (San Diego, CA, USA) included anti-CD3 (clone OKT3), anti-CD14 (clone M5E2), anti-CD56 (clone HCD56), anti-NKG2D (clone 1D11), anti-CD57 (clone HCD57), anti-CD11b (clone M1/70), anti-CD69 (clone FN50), anti-Tim3 (clone F38-2E2), anti-PD-1 (clone EH12.2H7), and anti-CD95 (clone DX2). Antibodies from BD Biosciences (San Jose, CA, USA) included anti-CD16 (clone 3G8) and anti-CD94 (clone HP-3D9). Antibodies from R&D Systems included anti-NKG2A (clone 131411) and anti-KIR2DL2/DL3/DS2 (clone 180704). Antibodies from Beckman Coulter (Indianapolis, IN, USA) included anti-KIR3DL1/DS1 (clone Z27.3.7) and anti-KIR2DL1/DS1 (clone EB6B). All panels also included the Fixable Blue Dead Stain (Life Technologies, Carlsbad, CA, USA) as a viability dye. K562 cells were stained with antibodies to PD-L1 (BioLegend, clone 29E.2A3) and Gal-9 (BioLegend, clone 9M1-3) with DAPI (BioLegend) as a viability dye. After staining, cells were fixed with 2% paraformaldehyde and analyzed using the BD Fortessa instrument (BD, Franklin Lakes, NJ, USA) and FlowJo software (TreeStar, Ashland, OR, USA).

### Calculation of Per-Cell Fold Expansion

The proportion of total live cells belonging to each NK subset was calculated using FlowJo software and then multiplied by the number of PBMCs added to start the culture at day 0 to determine the absolute number of cells in each subset. On day 12, the proportion of total NK cells belonging to each NK subset was calculated and multiplied by the number of NK cells isolated following expansion to give the absolute number of cells in each subset. Fold change for each subset was calculated as the absolute number of cells present following expansion divided by the absolute number of cells in the starting culture. Similar calculations were employed for T cells and monocytes.

### Degranulation/Intracellular Cytokine Staining

Cytokine production and degranulation were analyzed by flow cytometry. NK cells were seeded in a 96-well round bottom plate either alone (control) or together with K562 wild-type (WT) cells at a 1:1 ratio and incubated with anti-CD107a antibody (clone H4A3, BioLegend) for 1 h at 37°C/5% CO_2_, followed by Brefeldin A (10 µg/mL, Sigma, St. Louis, MO, USA) for an additional 3 h. Cells were then washed and stained with Fixable Blue Dead Stain and surface antibodies to CD3, CD56, and CD16. Cells were then fixed, permeablized with 1× Permwash (BioLegend), and stained intracellularly for GzmB (clone GB11, BD Biosciences), perforin (clone B-D48, BioLegend), and IFNγ (clone 4S.B3, BioLegend). For samples with antibody blockade, NK cells were incubated with antibodies (10 µg/mL in PBS with 2% bovine serum albumin, LEAF purified, same clones as above, BioLegend) for 45 min, washed, then mixed with K562 cells. Samples were analyzed using the BD Fortessa instrument and FlowJo software.

### Chromium Release Cytotoxicity Assays

K562 WT cells (target) were labeled with ^51^Cr (5 mCi/mL; Perkin Elmer, Waltham, MA, USA) for 24 h and washed. NK cells (effector) were cocultured with ^51^Cr-labeled K562 cells at 25:1, 10:1, 5:1, and 1:1 Effector:Target (E:T) ratios in 96-well plates for 4 h at 37°C, 5% CO_2_ as previously described (25). Fifty microliters of supernatant were harvested and dispensed in corresponding wells of a LumaPlate (Perkin Elmer). LumaPlates were allowed to dry overnight at room temperature and were then analyzed on a TopCount NXT (Perkin Elmer) to determine the amount of ^51^Cr released from lysed K562 target cells in counts per minute (cpm). Maximum chromium-51 release was determined by incubation of K562 target cells in 2% SDS solution; spontaneous release was obtained by incubation of target cells in the absence of effectors (media only). The mean percent of cytotoxicity was calculated using the following formula: ((cpm in experimental release − cpm in spontaneous release)/(cpm in maximum release − cpm in spontaneous release)) × 100. Differences between day 0 and day 12 samples were tested for significance using a two-way ANOVA.

### Nanostring (NS) Gene Expression Analysis

Donor-matched NK cells were isolated before or following expansion, rested in NK media overnight, and collected at 10,000 cells/μL in RLT buffer (Qiagen, Germantown, MD, USA) supplemented with beta-mercaptoethanol (Bio-Rad, Hercules, CA, USA). RLT lysate was then used directly in the Human Inflammation Panel (Nanostring, Seattle, WA, USA) per the manufacturer’s protocols. Nanostring nSolver Advanced Analysis software was used to normalize gene expression across samples, and *p* values of expression changes between day 0 and day 12 samples were calculated by paired Student’s *t*-test with a Benjamini–Yekutieli multiple comparisons adjustment.

### Quantitative PCR of Tbet and Eomes

Total RNA was isolated from excess RLT lysates collected for NS analysis using the RNeasy kit (Qiagen), then cleaned and concentrated using the RNeasy Min-Elute kit (Qiagen). An equivalent amount of total RNA (approximately 500 ng per sample) was reverse transcribed to cDNA with the SuperScript III kit (Invitrogen, Carlsbad, CA, USA). Quantitative PCR was performed on the cDNA with SYBR Green Master Mix (ThermoFisher) on the Bio-Rad CFX96 thermocycler. The 2^−ΔΔCt^ method was used to calculate the fold change between day 0 and day 12 samples, including TBP as the housekeeping gene. Primer sequences (written 5′–3′): TBP 5′: GAGCTGTGATGTGAAGTTTCC, TBP 3′: TCTGGGTTTGATCATTCTGTAG, Tbet 5′: GCTCACAAACAACAAGGGGG, Tbet 3′: TATGCGTGTTGGAAGCGTTG, Eomes 5′: CGCCACCAAACTGAGATGAT, and Eomes 3′: TTGTTGCCCTGCATGTTATTGT.

### Reproducibility and Statistics

All experiments were performed on at least three independent healthy donors. Unless otherwise noted, paired Student’s *t*-tests or one-way ANOVAs with Sidak’s multiple comparisons test were use to test for significance, as appropriate, using Prism software (GraphPad Software, San Diego, CA, USA).

## Results

Previous studies have demonstrated the ability of expanded NK cells to efficiently kill target cells (12–14, 26, 27), suggesting a mature and terminally differentiated phenotype. However, the degree to which an expanded NK product resembles NK cells found in peripheral circulation is currently unknown. It is likely that the phenotype of NK cells following expansion will at least in part predict patient response, as has been seen in adoptively transferred T cells (28–30). Therefore, we sought to define NK phenotype following expansion in the presence of IL-2 and K562 cells expressing membrane-bound IL-15 and costimulatory 4-1BBL. This feeder line has been used to expand NK cells that have been used to successfully treat patients in clinical trials (31, 32).

**Figure 1:**
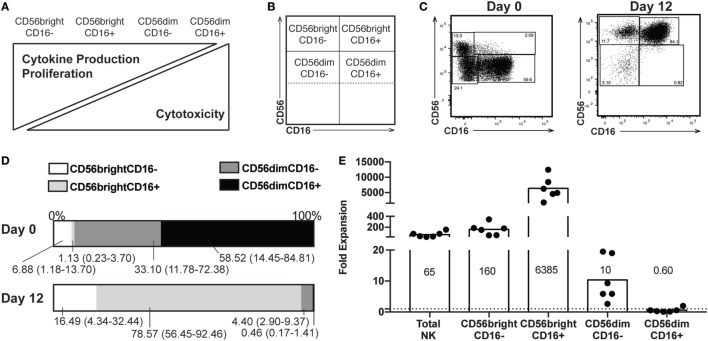
Proportions of natural killer (NK) subsets following expansion. **(A)** Spectrum of NK functions and maturity based on expression of CD56 and CD16. **(B)** Representation of where subsets appear on flow plots of NK cells. **(C)** Distribution of CD56 and CD16 expression by NK cells from a representative donor before (day 0) and after (day 12) expansion. Percentage of NK cells in each subset is included in each gate. **(D)** Percentages of each subset at day 0 (top) and day 12 (bottom). Labels for each subset indicate a mean and range (*n* = 6). **(E)** Expansion of total NK cells and each subset on a per-cell basis. The mean fold change is indicated for each subset (*n* = 6).

We first characterized NK cell subsets by CD16 and CD56 expression, using the gating strategy outlined in Figure S1 in Supplementary Material. NK cells exist on a spectrum of maturation, with less mature cells expressing high levels of CD56 responsible for the majority of cytokine production upon activation. As NK cells mature, they reduce CD56 levels and acquire CD16 expression, enabling efficient antibody-dependent cell cytotoxicity, and are the main cytotoxic population (Figures 1A,B). We performed flow cytometry to analyze NK cells from healthy donors both before (day 0) and after (day 12) expansion of NK cells (representative donor, Figure 1C; gating strategy in Figure S1 in Supplementary Material). Most notably, in addition to an increased proportion of the total NK cells (from approximately 10% before expansion to >90%) falling in a CD56 bright subset, CD56 staining intensity increases across all NK cell populations. CD56dim cells are concomitantly decreased in the day 12 cells, as illustrated in Figure 1D. On a per-cell basis, the four subtypes of NK cells varied in their fold expansion, with total NK cells expanding approximately 65-fold, consistent with previous reports (13, 14), the CD56bright CD16+ subset expanding more than 6,000-fold, and the amount of CD56dim CD16+ after 12 days of culture was 40% less than the number input initially (Figure 1E). Furthermore, we observed no monocyte expansion and minimal combined NKT and T cell expansion (average fivefold expansion of total CD3+ cells) (Table S1 in Supplementary Material). Similar expansion of CD3+ cells was seen by other investigators who used a high concentration of IL-2 (14). Because of the low number of certain cell subsets, subsequent analysis delineates expanded NK cell subsets based only on CD56 expression.

**Figure 2:**
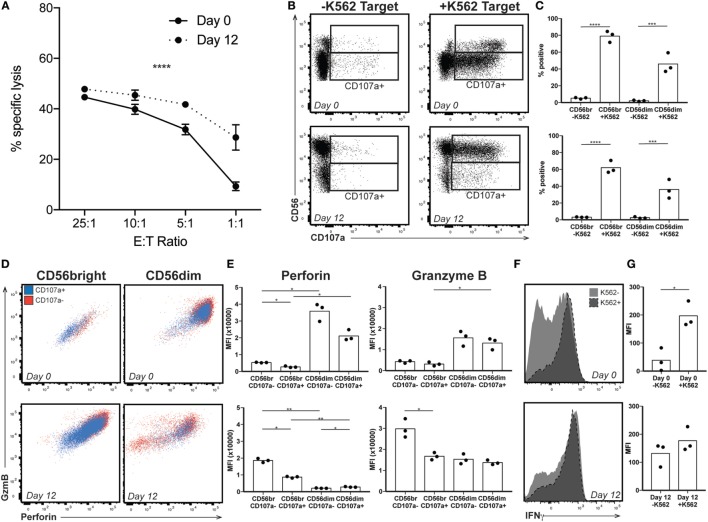
Expanded natural killer (NK) cell response to target. **(A)** By chromium release assay, NK cells lyse target more efficiently at low E:T ratio following expansion (representative donor). Statistical significance was assessed by two-way ANOVA (*p < 0.05, **p < 0.01, ***p < 0.001, and ****p < 0.0001). **(B)** Day 0 (top) and day 12 (bottom) NK cells from a representative donor were incubated without (−K562) or with (+K562) target cells and degranulation monitored by staining for the endosomal protein CD107a. Gates indicate CD107a positivity for CD56bright and CD56dim populations. **(C)** Quantification of degranulation by CD56 expression in response to K562 target at day 0 (top) or day 12 after expansion (bottom). Statistical significance was assessed by one-way ANOVA. **(D)** Granzyme B (GzmB) and perforin content by CD56 expression in the presence of K562 target cells in a representative donor. Cells that have released endosomal contents (CD107a+) are represented in blue, while those that did not degranulate (CD107−) are in red. **(E)** Quantification of perforin and GzmB MFI in the presence of K562 target cells. Day 0 NK cells are shown on top, and expanded day 12 NK cells on the bottom. Statistical significance was assessed by one-way ANOVA. **(F)** Interferon-γ expression by total NK cells from a representative donor in the presence or absence of K562 target cells before (top) or after (bottom) expansion. **(G)** Quantification of IFNγ MFI in response to K562 target cells. Day 0 NK cells are shown on top, and expanded day 12 NK cells on the bottom. Statistical significance was assessed by Student’s *t*-test (*p < 0.05, **p < 0.01, ***p < 0.001, and ****p < 0.0001).

High levels of CD56 expression on the surface of NK cells is associated with reduced cytotoxic activity (33). Therefore, we wanted to confirm that the expanded NK cells were functionally similar to those used in previous studies, which have found increased cytotoxicity following expansion (12–14, 26, 27, 34, 35). Using a chromium release assay (CRA), we confirmed that by day 12 of expansion, NK cells were more efficient at lysing K562 target cells (representative donor, Figure 2A). This suggested that the enriched CD56bright population, generally considered more involved in cytokine production than cytotoxicity, must be playing a significant part in killing the chromium-labeled cells. We therefore performed a degranulation assay, which measures release of cytotoxic granules and externalization of the endosomal protein CD107a. In response to exposure to target cells, both CD56bright and CD56dim subsets externalized CD107a (Figures 2B,C). However, in freshly isolated NK cells, the low basal perforin and GzmB content in the CD56bright cells suggest a limited capacity to lyse target cells despite evidence of degranulation, a phenomenon supported by CRA data at lower effector to target ratios. CD56dim cells express high levels of perforin and GzmB; staining is decreased, suggesting degranulation, upon exposure to target cells (Figures 2D,E, top). By contrast, at day 12, staining for perforin and GzmB is high in CD56bright cells and decreases following target exposure (Figures 2D,E, bottom); CD56dim cells do not appear to be releasing significant cytotoxic granules. Finally, although cytokine production and cytotoxicity are functions performed by different subsets in peripheral blood NK cells, by day 12, the CD56bright subset is still responsible for IFNγ production upon target exposure on top of its role in cytotoxicity (Figures 2F,G), suggesting that the expanded NK cell product is comprised of a cell type that phenotypically resembles immature NK cells with high proliferative and cytokine secretory capacity, and functionally retains the properties of an immature NK cell, but with enhanced cytotoxic capability.

**Figure 3:**
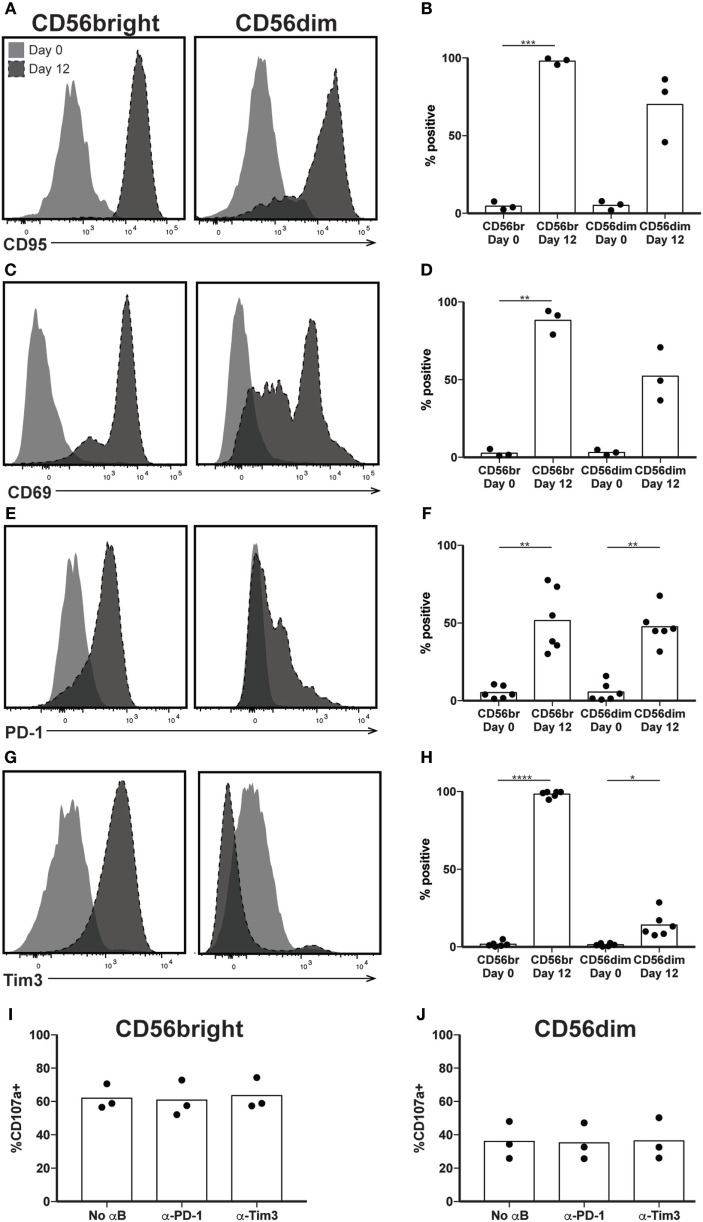
Expression of exhaustion markers following natural killer (NK) expansion. **(A–H)** Overlays of NK cells from a representative donor before (day 0) and after (day 12) expansion, by CD56bright (left) and CD56dim (right), for CD95 **(A)**, CD69 **(C)**, PD-1 **(E)**, and Tim3 **(G)**. Quantification of percent cells positive at day 0 and day 12 for CD95 **(B)**, CD69 **(D)**, PD-1 **(F)**, and Tim3 **(H)**. Statistical significance was assessed by one-way ANOVA (*p < 0.05, **p < 0.01, ***p < 0.001, and ****p < 0.0001). **(I,J)** Percentage of expanded CD56bright **(I)** or CD56dim **(J)** NK cells that degranulated (CD107a+) in the presence of blocking antibodies to PD-1 or Tim3. Statistical significance was assessed by one-way ANOVA, and no differences seen.

Our results so far indicate that by day 12, a highly cytotoxic subset of NK cells has expanded, although its function appears decoupled from its CD56 expression, a phenomenon seen in NK cells cultured in IL-2 and/or IL-15 (12, 36, 37). Given the concern of T cell persistence *in vivo* following *ex vivo* expansion, and the potential for the sustained activation to contribute to exhaustion, we examined canonical markers of T cell activation and exhaustion on the surface of expanded NK cells, all of which were expressed on 5% or fewer NK cells before expansion. Expression of CD95 occurs in T and NK cells upon their activation, and following expansion, nearly 100% of CD56bright and >50% of CD56dim NK cells are CD95+ (Figures 3A,B). Similarly, the C-type lectin CD69 serves as a marker of nonspecific activation in response to various stimuli (38) and its upregulation is observed after expansion on both CD56bright and CD56dim NK cells (Figures 3C,D). Unlike CD69 expression on CD8+ T cells, however, CD69 expression does not wane following initial upregulation (11), and therefore is not exclusively a marker of early activation. By day 12, the well-characterized T cell exhaustion marker PD-1 increases from about 5% to approximately 50% expression on both CD56bright and CD56dim subsets (Figures 3E,F). Tim3, another marker of T cell exhaustion, increases on CD56bright cells from 2% at day 0 to 100% by day 12 (Figures 3G,H), and to 14% on CD56dim NK cells. K562 target cells express the PD-1 ligand PD-L1 and the Tim3 ligand Gal-9 (Figure S2 in Supplementary Material). Blockade of either PD-1 or Tim3 did not decrease degranulation, however (Figures 3I,J), suggesting that expanded NK cells were not functionally exhausted.

**Figure 4:**
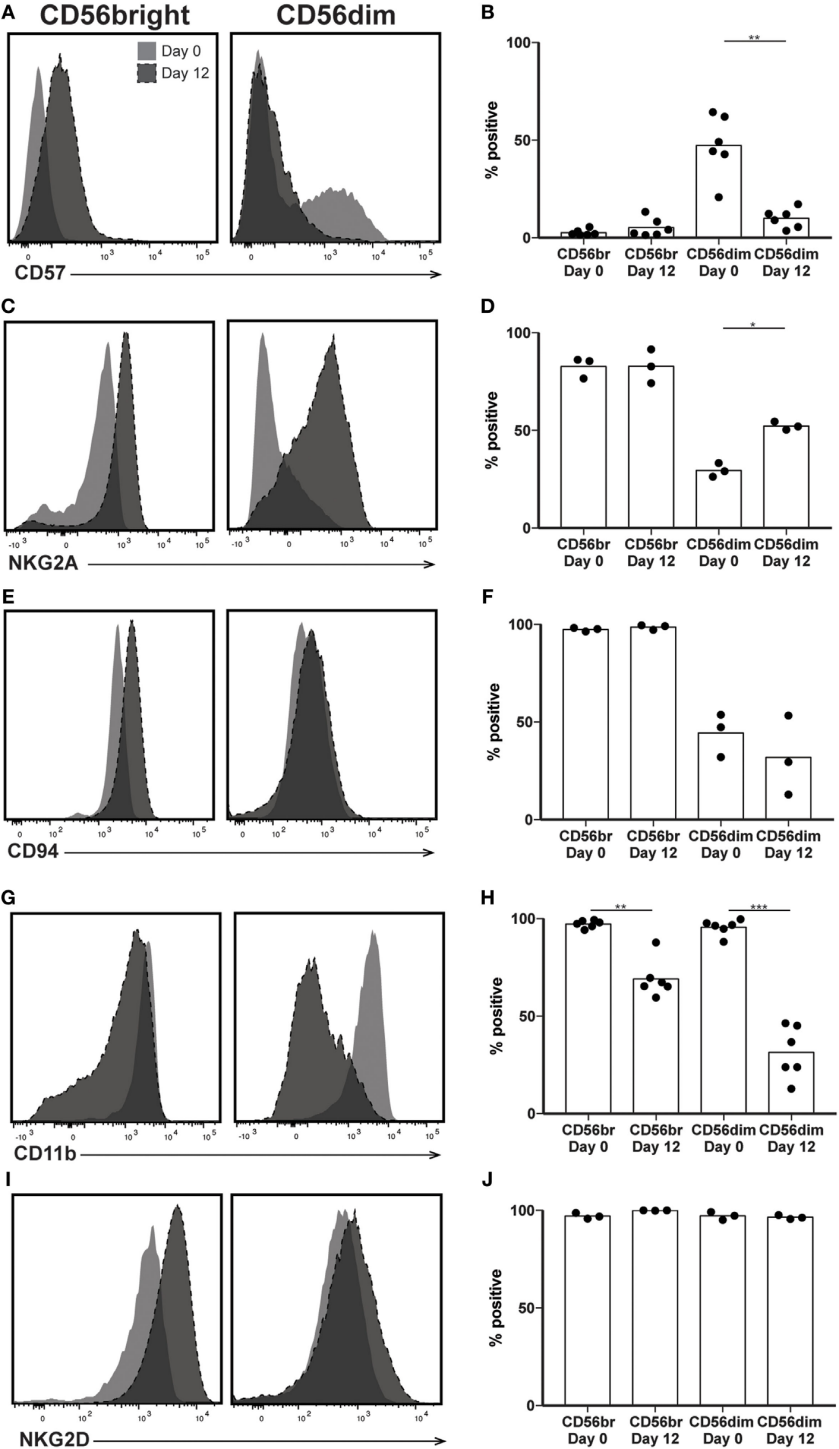
Expression of maturity markers following natural killer (NK) expansion. **(A–J)** Overlays of NK cells from a representative donor before (day 0) and after (day 12) expansion, by CD56bright (left) and CD56dim (right), for CD57 **(A)**, NKG2A **(C)**, CD94 **(E)**, CD11b **(G)**, and Tim3 **(I)**. Quantification of percent cells positive at day 0 and day 12 for CD57 **(B)**, NKG2A **(D)**, CD94 **(F)**, CD11b **(H)**, and Tim3 **(J)**. Statistical significance was assessed by one-way ANOVA (*p < 0.05, **p < 0.01, ***p < 0.001, and ****p < 0.0001).

In addition to CD56, several other surface markers and inhibitory receptors have been associated with the state of NK cell maturity. Despite significant CD56 and IFNγ production by the predominant NK subsets at day 12, increased cytotoxicity and expression of exhaustion markers suggest that expanded NK cells would express other proteins present on mature NK cells. Therefore, we expected most expanded NK cells to express CD57, a marker of highly cytotoxic, terminally differentiated NK cells (25). Although there was not a significant increase in CD57 expression by day 12 when comparing CD56bright subsets, comparison of predominant NK cell populations before and after expansion revealed a decrease in the number of NK cells expressing CD57. At day 0, an average of 47% of CD56dim NK cells express CD57, while at day 12, only about 5% of CD56bright cells express CD57 (Figures 4A,B). We also looked at expression of NKG2A, an inhibitory receptor for HLA-E expressed by immature NK cells, expecting the opposite trend of CD57 expression, which was lost upon expansion. We were surprised to see retention of NKG2A in the CD56bright subset following expansion, and a concomitant increase in expression by the CD56dim subset (Figures 4C,D). The percentage of cells expressing CD94, the co-receptor for NKG2A, also remained constant throughout expansion (Figures 4E,F). The integrin CD11b (ITGAM) is generally expressed on most NK cells found in peripheral circulation but absent in immature populations found in particular biological niches in both mice and humans (39, 40). Consistent with these observations, following >95% expression at day 0 in both subsets, CD11b expression after expansion drops to 69 and 31% on CD56bright and CD56dim subsets, respectively (Figures 4G,H). Finally, expression of NKG2D, an activating receptor that binds to the stress ligands MICA/B and ULBP1–6, remains near 100% in all NK cell samples (Figures 4I,J).

**Figure 5:**
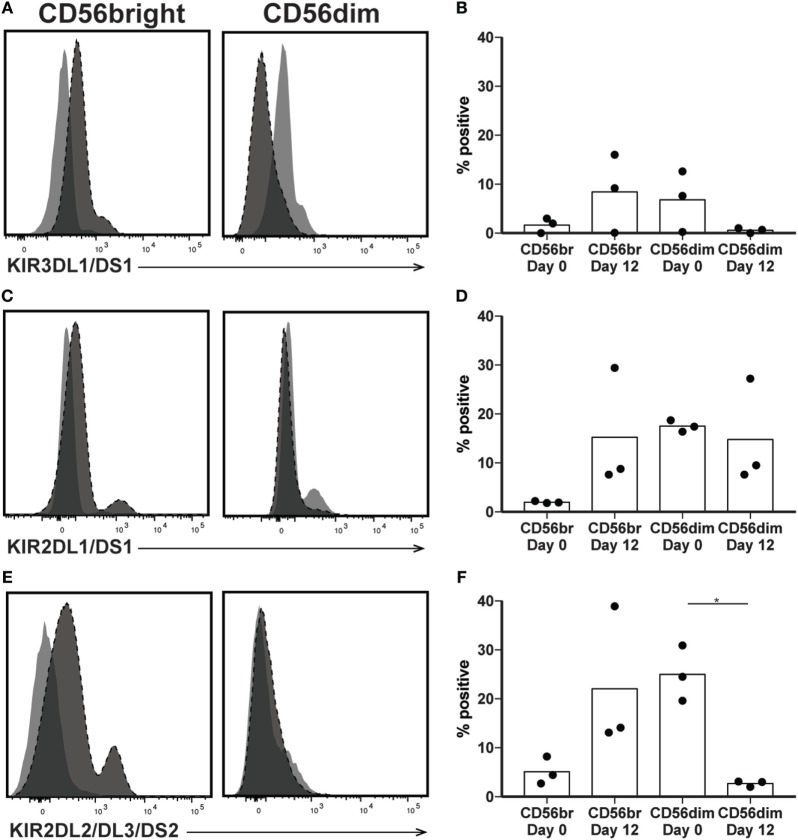
Expression of killer immunoglobulin receptors following natural killer (NK) expansion. **(A–F)** Overlays of NK cells from a representative donor before (day 0) and after (day 12) expansion, by CD56bright (left) and CD56dim (right), for KIR3DL1/DS1 **(A)**, KIR2DL1/DS1 **(C)**, or KIR2DL2/DL3/DS2 **(E)**. Quantification of percent cells positive at day 0 and day 12 for KIR3DL1/DS1 **(B)**, KIR2DL1/DS1 **(D)**, or KIR2DL2/DL3/DS2 **(F)**. Statistical significance was assessed by one-way ANOVA (*p < 0.05, **p < 0.01, ***p < 0.001, and ****p < 0.0001).

**Figure 6:**
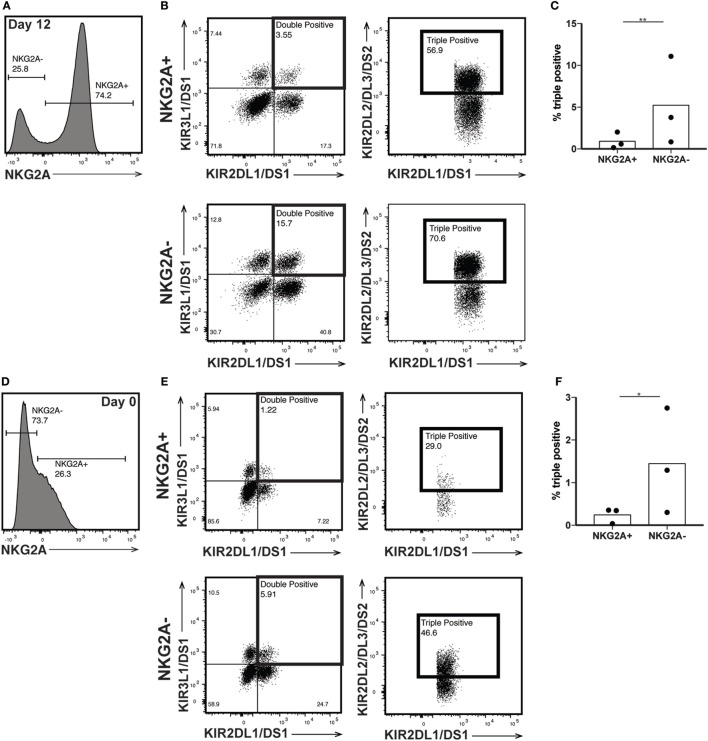
Killer immunoglobulin-like receptor (KIR) expression is enriched in NKG2A- natural killer (NK) cells. **(A)** Day 12 expanded CD56bright NK cells were gated on NKG2A expression. **(B)** NKG2A+ (top) and NKG2A− (bottom) cells were interrogated for their expression of both KIR2DL1/DS1 and KIR3DL1/DS1 (left). KIR2DL2/DL3/DS2 expression was examined on the double positive cells (right). **(C)** Percent of total NKG2A+ or NKG2A− cells that are positive for all three KIRs. Statistical significance was assessed by Student’s *t*-test. **(D)** Day 0 CD56dim NK cells were gated on NKG2A expression. **(E)** NKG2A+ (top) and NKG2A− (bottom) cells were interrogated for their expression of both KIR2DL1/DS1 and KIR3DL1/DS1 (left). KIR2DL2/DL3/DS2 expression was examined on the double positive cells (right). **(F)** Percent of total NKG2A+ or NKG2A− cells that are positive for all three KIRs. Statistical significance was assessed by Student’s *t*-test (*p < 0.05, **p < 0.01, ***p < 0.001, and ****p < 0.0001).

The killer immunoglobulin-like receptors (KIRs) are a family of NK receptors whose activating or inhibitory effect on cytotoxicity is determined by the cytoplasmic domain found on identical surface proteins that are acquired late during NK cell maturation (41). Given our conflicting phenotypic and functional results, we were uncertain whether to expect increased KIR expression, as would be associated with differentiation, or decreased KIR expression, more consistent with a lack of maturity. KIR expression increased in percent positivity (to approximately 8, 15, and 22% positive on KIR3DL1/DS1, KIR2DL1/DS1, and KIR2DL2/DL3/DS2, respectively) on CD56bright cells by day 12 of expansion, reaching expression similar to that found on CD56dim cells in unexpanded cultures at day 0 (Figures 5A–F), although increases were not statistically significant. However, even within the expanded population of CD56bright NK cells, there remains at least some degree of maturation heterogeneity: when gated on NKG2A positive or negative cells, which are typically considered less or more mature, respectively, NK cells expressed all three KIR subgroups on an average of fewer than 1% of cells, versus more than 5% of NKG2A- cells (Figures 6A–C). A similar trend is seen for the day 0 CD56dim cells, although overall KIR percent triple positive is lower (Figures 6D–F). This is consistent with the enhanced cytotoxicity observed in the expanded cells, which appear to have a disparate relationship between maturity and cytolytic capacity than is typically described for NK cells based on CD56 expression. Collectively, these data suggest that CD56 expression is uncoupled from function in *ex vivo*-expanded NK cells.

**Figure 7:**
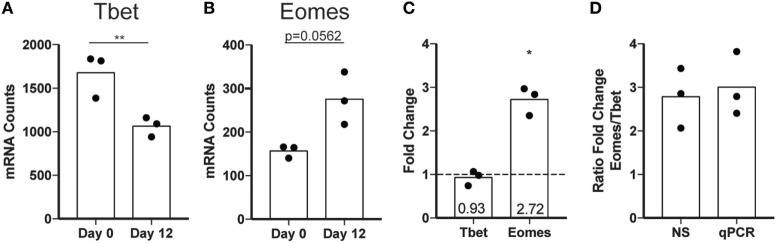
Transcription factor expression changes following expansion. **(A)** Tbet mRNA expression in total natural killer (NK) cells assessed by nanostring (NS) at day 0 or day 12. Statistical significance was assessed by Student’s *t*-test. **(B)** Eomes mRNA expression in total NK cells assessed by NS at day 0 or day 12. **(C)** Fold change by qPCR calculated by the ΔΔCt method for Tbet and Eomes. Statistical significance was assessed by Student’s *t*-test (*p < 0.05, **p < 0.01, ***p < 0.001, and ****p < 0.0001). **(D)** Ratio of the fold change for Eomes relative to the fold change in Tbet as calculated for NS and qPCR.

To assess expression of maturation markers independent of CD56 surface expression, we evaluated the expression of transcription factors critical to NK cell gene expression in different stages of maturation. Differentiation of NK cells in peripheral circulation is driven largely by the reciprocal expression of two transcription factors, Eomes and Tbet. Mature NK cells have a higher ratio of Tbet to Eomes than immature NK cells, and the increase in Tbet throughout maturation is implicated in the acquisition of cytotoxic functions (42). To determine whether *ex vivo* expansion of NK cells impacts the relative expression of Eomes and Tbet, we performed NS analysis of freshly isolated NK cells before or after expansion using a panel of genes associated with inflammation (Table S2 in Supplementary Material). We found a decrease in Tbet (Figure 7A) and an increase in Eomes (Figure 7B) following expansion, supporting the hypothesis that *ex vivo*-expanded NK cells represent a less differentiated NK cell population with potentially extended longevity and responsiveness to various activating stimuli *in vivo*. This was confirmed by qPCR of Tbet and Eomes (Figure 7C). Although the reduction in Tbet in day 12 cells was smaller as measured by qPCR relative to NS, the increase in Eomes/Tbet ratio is similar (Figure 7D).

In our NS analysis, we confirmed several trends we had seen by flow cytometry, including upregulation of CD56, IFNγ, and specific KIR subgroups following expansion. We also found altered expression of several genes we had not included in our flow cytometry panels, but are consistent with acquisition of highly cytotoxic, yet less traditionally mature phenotype. We saw 54-fold upregulation of Granzyme K and 3.5-fold upregulation of Granzyme A, alongside a 2-fold decrease in GzmB (Table S2 in Supplementary Material), consistent with previous reports of less differentiated CD56bright cells relying on alternative granzymes (43). We also saw a striking increase in several chemokine receptors, CCR1, CCR2, CCR5, CCR6, CXCR3, and CXCR6 (Table S2 in Supplementary Material), suggesting that expanded NK cells may be better able to localize to tumors, many of which overexpress the cognate chemokines (44). Therefore, we propose that the current method for *ex vivo* expansion of NK cells selects for a potent subset capable of proliferation, cytokine production, cytotoxicity, and persistence, making this an ideal adoptively transferred cell population for the treatment of patients with cancer.

## Discussion

Natural killer cells play an important role in immunosurveillance, and their unique ability to eliminate transformed cells without the requirement of a tumor-specific antigen makes them an attractive cell type for the next generation of cancer immunotherapeutics. Following the initial reports of solid tumor regression following high-dose systemic IL-2 as early as 1985 (45), subsequent efforts demonstrated NK cell efficacy in eliminating both solid and hematologic cancers in animal models and clinical trials (31, 32, 46–48). However, like antigen-specific T cells, NK cells have relatively low abundance in peripheral blood, requiring *ex vivo* expansion before adoptive transfer.

As NK immunotherapy is increasingly used in the clinic, an improved evaluation of the expanded cell product with respect to phenotype, functions, and persistence *in vivo* may be of considerable benefit to iterative improvements in selection of patients, cell subsets, and/or *ex vivo* expansion protocols. Recent assessments of CAR T cell products that use well-defined phenotypes to evaluate the ratios of short-lived effectors and persistent memory T cells may be predictive of patient response (29). Phenotypes and functional capacities of *ex vivo*-expanded NK cells, however, are less well understood. To this end, we analyzed the phenotypes and functions of NK cells expanded using a promising method in which PBMCs are cocultured with K562 cells engineered to express the costimulatory molecule 4-1BBL and membrane-bound IL-15, in the presence of high-dose soluble IL-2 developed by Imai et al. (12).

Because several reports (12–14, 26, 27, 34, 35) demonstrated the enhanced cytolytic functions of *ex vivo*-expanded NK cells that are typically associated with more mature, CD56dim NK cells, we expected that *ex vivo*-expanded NK cells would predominantly comprised of a fully differentiated subset characterized by low surface CD56, expression of CD11b and CD57, proteins associated with terminal differentiation, and loss of the inhibitory receptor NKG2A. Relative to donor-matched NK cells that had not undergone *ex vivo* expansion, however, the majority of expanded NK cells had CD56 expression that was higher than was detected on any subset before expansion. This is consistent with previous reports showing increased CD56 on NK cells that have been *ex vivo* expanded (12). Furthermore, the CD56bright population is enriched in patients with chronic infections (49, 50) or autoimmune conditions (51), suggesting increased CD56 expression may be related to chronic stimulation. However, high CD56 expression on expanded NK cells was accompanied by an increased proportion of cells expressing NKG2A, a protein generally associated with immaturity, and negative for markers of terminal differentiation, including CD11b and CD57. Phenotypically, these results suggested that expanded NK cells were not terminally differentiated, a conclusion supported by the increased expression of the Eomes and reduced expression of Tbet, the transcription factors responsible for the spectrum of NK cell differentiation.

Like T cells, immature NK cells are generally less capable of recursive killing than their terminally differentiated counterparts. Since T cells and NK cells share many of the molecular programs that drive terminal differentiation, there were similar concerns for impairment of NK cell functions following extensive *ex vivo* culture and expansion. Paradoxically, we and others found that *ex vivo*-expanded NK cells have an enhanced ability to eliminate tumor cells (12–14, 26, 27, 34, 35). Interestingly, the phenotypically immature, predominant CD56bright population degranulates upon exposure to target cells, a function that is generally associated with terminally differentiated, CD56dim/CD11b+ NK cells. However, like NK cell subsets isolated from patients, the CD56bright population retained its proliferative capacity and ability to produce IFNγ in response to target cells. In contrast to NK cells isolated from peripheral blood, it appears that following *ex vivo* expansion, the delineation of NK cell subsets that are dedicated to cytotoxic function or cytokine production is not associated with the amount of CD56 or other maturation marker expression. Together, these data suggest an uncoupling of phenotypic markers of maturation and canonical functions of NK cell subsets in *ex vivo*-expanded NK cells.

Although NK cells do not express antigen-restricted receptors in the manner of T or B cells, each NK cell does express an array of activating and inhibitory KIRs; the complement of KIRs expressed by any NK cell develops stochastically during maturation and allows significant diversity in the responsiveness of each NK cell to a particular signal (41). Because the expanded NK cells appear more homogeneous than peripheral blood NK cells, as evidenced by both IFNγ production and cytotoxicity occurring in the same cells, we expected that this would be associated with the predominance of a clonal population with uniform KIR expression. By contrast, we found that expanded NK cells had both positive and negative populations of all KIRs evaluated, and that expression of one KIR did not appear to predict expression of another. This is consistent with a report that culture with IL-2 and IL-15 induced expression of an assortment of KIRs on CD56bright cells (36). Expanded NK cells maintained the KIR expression diversity seen in freshly isolated NK cells, suggesting that the expansion protocol does not enhance NK cell clonal dominance. Furthermore, the increased number of KIRs expressed by any single NKG2A negative NK cell suggests that expanded NK cells retain a spectrum of mechanisms of activation. Retention of KIR diversity may be important because homogeneity in KIR expression could limit efficacy, or exert selective pressure to reduce expression of NK cell activating proteins on the surface of tumor cells, contributing to tumor immune evasion through immunoediting.

T cell expansion protocols that use repeated stimulation and sustained cytokine exposure can result in T cell anergy and replicative senescence of the terminally differentiated effector cells. T cells expanded in these conditions can therefore undergo the phenotypic and functional impairments that are incurred during chronic infections, reducing efficacy in tumor cell elimination following adoptive transfer (52–54). The development of immune checkpoint blockade, which disrupts the interaction between PD-1 and CTLA4 on T cells with their respective ligands to reverse exhaustion caused by chronic antigen exposure, has been an important addition to the arsenal of treatments for several cancers, including melanoma, lung cancer, and bladder cancer (55). The molecular mechanisms underlying NK cell exhaustion are not as clearly defined as they are for T cells, but both PD-1 and Tim3 have been explored as markers of functional NK cell exhaustion. PD-1 expression on NK cell lines can induce exhaustion (56) but Tim3+ NK cells stimulated with IL-12/IL-18 have high cytotoxicity and cytokine production, suggesting Tim3 may be a maturation marker (57). Increased expression of PD-1 and Tim3 has been found on NK cells isolated from cancer patients (24, 56, 58) and patients with chronic viral infections (59). In spite of the increased expression of PD-1 and Tim3 on *ex vivo*-expanded NK cells, expanded NK cells had enhanced cytotoxic activity and maintained the potential for IFNγ production. Similarly, blockade of PD-1 or Tim3 failed to enhance degranulation of NK cells in response to target cells. Given the dissociation of surface marker expression and NK cell functions observed in *ex vivo*-expanded NK cells, it is possible that PD-1 and Tim3 expression on expanded NK cells do not have the same functional consequences as they do in T cells or resting NK cells. It is also possible that expanded NK cells can resist PD-1 mediated functional impairments, which are driven by PI3K/Akt pathway repression (60, 61), and may be overridden by the constitutive PI3K activating IL-15 signal incurred during expansion. Tim3 expression is also driven by IL-15 and cytokine stimulation may override any weak inhibitory effect of Tim3 (57). Furthermore, the observed increase in Eomes expression may play a role in preventing functional exhaustion of expanded NK cells (23).

Because NK cells are rare in peripheral blood, the primary goal of *ex vivo* expansion protocols is to obtain sufficient numbers for use in the clinic. In recent years, expansion of NK cells using only cytokines has been largely supplanted by the use of allogeneic feeder lines, including allogeneic PBMCs, lymphoblastoid cell lines, and K562 [reviewed in Ref. (62)]. K562 cells that have been genetically modified to express cytokines and costimulatory factors for NK cells have shown promise for both their robust expansion and favorable cytotoxicity toward cancer cells. These include the K562-mb15-4-1BBL cells we used in this study and have been successfully employed in the clinic (31, 32), as well K562-4-1BBL cells also expressing membrane-bound IL-21 (63) or the NKG2D ligand MICA (64). In particular, Denman and colleagues’ development of an expansion protocol using K562 cells expressing membrane-bound IL-21, a common γ-chain cytokine like IL-2 and IL-15, improved the expansion and cytokine production of NK cells relative to those expanded with K562-mbIL15 while maintaining a similar phenotype and cytotoxic capacity (63). Notably, IL-21-expanded NK cells may represent the latest improvement in the iterative process of developing an effective NK cell expansion protocol, showing efficacy in mouse models of neuroblastoma (65). Although we did not test these cells directly, we expect that because IL-15 and IL-21 share many functional similarities and stimulate overlapping signaling cascades, many of our findings surrounding the uncoupling of function from phenotype will also apply to K562-4-1BBL-mb21-expanded NK cells.

Alongside robust expansion, an important consideration in the development of novel NK cell expansion protocols for use in the clinic is the safety profile of the adoptively transferred product. Because NK expansion protocols frequently include factors such as high-dose IL-2, IL-15, and IL-21 that also stimulate proliferation of T cells, graft-versus-host disease is a particular concern. Therefore, elimination of as many CD3+ T cells as possible is a key step in the certification of NK expansion protocols as GMP compliant. In our hands, CD3+ cells expanded an average of fivefold and represented an average of less than 30% in the final product on day 12, consistent with previous reports that have shown effective elimination of all but trace CD3+ cells by the CliniMACS system (66, 67). There is currently debate in the field about the relative merits of expanding from bulk PBMCs and eliminating contaminating CD3+ cells before infusion, versus expanding from purified NK cells and minimizing concerns about CD3+ contamination at the outset; a recent study found both methods to be functionally equivalent when using K562s modified to express an array of costimulatory molecules, with the advantage of the latter in driving down production costs (63). The presence of monocytes is known to aid in the activation of NK cells (68, 69), as do activated T cells (70). Therefore, development of genetically modified feeder cells that provide the support currently offered by monocytes and T cells may represent an opportunity to improve safety, cost, and fold expansion of NK cells for adoptive transfer.

Collectively, our results suggest that *ex vivo* expansion of NK cells using IL-2 and K562 cells engineered to express 4-1BBL and membrane-bound IL-15 produces a population of NK cells whose phenotype and function do not fit cleanly onto the spectrum of activation and maturity associated with peripheral blood NK cell subsets. Rather, expanded NK cells are capable of both IFNγ production and cytotoxicity and have phenotypic characteristics associated with both mature and immature NK cells. Notably, expanded NK cells have altered the expression of two transcription factors that influence differentiation, increasing expression of immaturity-associated Eomes while downregulating Tbet, which drives the terminal differentiation program in NK cells (71). It is unclear whether this represents de-differentiation of a mature, cytotoxic subset of NK cells, or the prolific emergence of an immature subset that, through *ex vivo* expansion by 4-1BBL/IL-15, acquires functions that are historically associated with terminally differentiated NK cells. Following administration to multiple myeloma patients, NK cells *ex vivo* expanded with K562-mb15-4-1BBL and high-dose IL-2 exhibit high CD56 expression and proliferate extensively (31). *In vitro*, culture of NK cells with IL-15 causes dilution of CFSE primarily in the immature CD56bright subset (36). This is consistent with reports of persistence of cord blood-derived CD56bright NK cells persisting for up to 2 years (72), as well as a cord. Furthermore, the CD56− CD16+ subset, a unique immature NK population found in cord blood, has also been found to persist following transplant to cancer patients and differentiate into cytotoxic CD56+ antitumor cells (73). Together, these data demonstrate that phenotypically immature NK cells, such as those generated by *ex vivo* expansion using K562-mb15-4-1BBL, can persist long term *in vivo*. Importantly, the resultant NK cell therapy product exhibits both enhanced antitumor activity and a relatively undifferentiated phenotype, which in T cells predicts the most robust and long-term antitumor responses following adoptive transfer. *Ex vivo* expansion with 4-1BBL/IL-15 may therefore generate a potent NK cell product with respect to cytotoxic capacity, versatility in mechanisms of activation, and longevity *in vivo* following adoptive transfer.

## Ethics Statement

All protocols have been reviewed and approved by the relevant institutional committees, including the Seattle Children’s Research Institute Institutional Biosafety Committee (Approval #1211) and Institutional Review Board (Approval #14412).

## Author Contributions

NAL, KH, CMC, and CAC conceived the study and designed experiments. MB and CMC developed protocols and provided technical support and troubleshooting. NL performed most experiments with help from KD and KH in particular, as well as HC. KW performed flow cytometry on K562 cells. NL, KH, and KM analyzed data. NL and CAC wrote the manuscript. All authors contributed to final editing.

## Conflict of Interest Statement

The authors declare that the research was conducted in the absence of any commercial or financial relationships that could be construed as a potential conflict of interest.
