# Supplementary material for: An Uncoupling of Canonical Phenotypic Markers and Functional Potency of Ex Vivo-Expanded Natural Killer Cells
Source: Front Immunol. 2018 Feb 2;9:150. doi: 10.3389/fimmu.2018.00150 (PMC5801405; doi:10.3389/fimmu.2018.00150)
Supplement: Supplementary file 1 [file Table_1.DOCX]

**Supplemental Table 1: Per cell fold enrichment of CD14+ and CD3+ cells in each of six healthy donors.**

|  | **FOLD CHANGE** |  |
| --- | --- | --- |
| **Donor:** | **CD14+ Monocytes** | **CD3+ T cells** |
| **A** | 0.015793085 | 6.950975163 |
| **B** | 0.428089196 | 1.761101771 |
| **C** | 0.037609451 | 9.705584705 |
| **D** | 0.003948047 | 2.662398001 |
| **E** | 0.002675317 | 2.495117285 |
| **F** | 0.005074766 | 7.571887582 |
|  |  |  |
| **mean** | **0.08219831** | **5.191177418** |
