# Supplementary material for: An Uncoupling of Canonical Phenotypic Markers and Functional Potency of Ex Vivo-Expanded Natural Killer Cells
Source: Front Immunol. 2018 Feb 2;9:150. doi: 10.3389/fimmu.2018.00150 (PMC5801405; doi:10.3389/fimmu.2018.00150)
Supplement: Supplementary file 2 [file Table_2.DOCX]

**Supplemental Table 2: Fold change of inflammation-related genes in NK cells following *ex vivo* expansion relative to non-expanded cells.** Bolded genes have been mentioned in the text.

| **Gene name** | **mRNA/probe ID** | **Fold Change** | **p value** |
| --- | --- | --- | --- |
| ABCB1 | NM_000927.3:3910 | 1.017620755 | 0.874 |
| ABL1 | NM_005157.3:3200 | 0.366021424 | 5.14E-05 |
| ADA | NM_000022.2:1300 | 2.128740365 | 0.019 |
| AHR | NM_001621.3:1900 | 0.687294348 | 0.0187 |
| APP | NM_000484.3:1725 | 1.828930179 | 0.174 |
| ARHGDIB | NM_001175.4:833 | 1.257013375 | 0.0678 |
| ATG10 | NM_001131028.1:985 | 0.81056512 | 0.43 |
| ATG12 | NM_004707.2:25 | 0.882702996 | 0.0518 |
| ATG16L1 | NM_198890.2:1975 | 1.095052471 | 0.519 |
| ATG5 | NM_004849.2:1104 | 0.981186983 | 0.864 |
| ATG7 | NM_001136031.2:810 | 0.426317446 | 0.0182 |
| B2M | NM_004048.2:235 | 0.69495911 | 0.0467 |
| BATF | NM_006399.3:293 | 0.341510064 | 0.0143 |
| BATF3 | NM_018664.2:770 | 0.285190929 | 0.00315 |
| BAX | NM_138761.3:342 | 1.333298677 | 0.11 |
| BCAP31 | NM_005745.7:495 | 1.016774673 | 0.866 |
| BCL10 | NM_003921.2:1250 | 0.708087719 | 0.0441 |
| BCL2 | NM_000657.2:947 | 0.524858342 | 0.0605 |
| BCL2L11 | NM_138621.4:257 | 0.89564567 | 0.518 |
| BCL3 | NM_005178.2:450 | 0.243163737 | 0.0051 |
| BCL6 | NM_001706.2:675 | 0.363493129 | 0.0362 |
| BID | NM_001196.2:1875 | 0.408951029 | 0.00999 |
| BST2 | NM_004335.2:560 | 1.273677475 | 0.111 |
| C14orf166 | NM_016039.2:210 | 0.796088099 | 0.00725 |
| C1QBP | NM_001212.3:745 | 0.543367431 | 0.00349 |
| C1R | NM_001733.4:760 | 4.28709385 | 0.00215 |
| C6 | NM_000065.2:3170 | 0.550570799 | 0.0568 |
| CASP1 | NM_001223.3:971 | 0.853226098 | 0.0647 |
| CASP2 | NM_032982.2:1710 | 0.66296288 | 0.00599 |
| CASP3 | NM_032991.2:685 | 0.991647993 | 0.502 |
| CASP8 | NM_001228.4:301 | 0.368567304 | 0.00229 |
| CCBP2 | NM_001296.3:1345 | 3.249009585 | 0.00142 |
| CCL3 | NM_002983.2:159 | 1.29145735 | 0.0783 |
| CCL4 | NM_002984.2:35 | 0.414659773 | 0.0376 |
| CCL5 | NM_002985.2:280 | 2.114036081 | 0.0901 |
| CCND3 | NM_001760.2:1215 | 0.922103118 | 0.538 |
| **CCR1** | **NM_001295.2:535** | **2.099433367** | **0.0237** |
| **CCR2** | **NM_001123041.2:743** | **24.7610399** | **0.0193** |
| **CCR5** | **NM_000579.1:2730** | **5.063026376** | **0.00354** |
| **CCR6** | **NM_031409.2:935** | **2.828427125** | **0.0166** |
| CCRL2 | NM_003965.4:1110 | 1.140763716 | 0.677 |
| CD160 | NM_007053.2:500 | 0.070805243 | 0.0511 |
| CD164 | NM_006016.4:2575 | 0.336808394 | 0.00995 |
| CD2 | NM_001767.3:687 | 1.965641197 | 0.135 |
| CD244 | NM_016382.2:1150 | 0.706616822 | 0.225 |
| CD247 | NM_198053.1:1490 | 0.681601304 | 0.0899 |
| CD274 | NM_014143.3:1245 | 0.931740429 | 0.489 |
| CD276 | NM_001024736.1:2120 | 2.345669898 | 0.0683 |
| CD3D | NM_000732.4:110 | 0.450625231 | 0.057 |
| CD3E | NM_000733.2:75 | 5.656854249 | 0.058 |
| CD3EAP | NM_012099.1:555 | 0.356012549 | 0.0288 |
| CD40 | NM_001250.4:1265 | 0.632439771 | 0.0307 |
| CD40LG | NM_000074.2:1225 | 1.673652485 | 0.236 |
| CD44 | NM_001001392.1:429 | 0.248273124 | 0.00436 |
| CD45R0 | NM_080921.3:258 | 29.85705573 | 0.00279 |
| CD45RA | NM_002838.4:258 | 0.025916236 | 0.00968 |
| CD45RB | ENST00000367367.1:131 | 1.614402149 | 0.213 |
| CD46 | NM_172350.1:365 | 0.705637922 | 0.00439 |
| CD48 | NM_001778.2:270 | 0.942000676 | 0.739 |
| CD53 | NM_001040033.1:835 | 0.740206649 | 0.175 |
| CD55 | NM_000574.3:101 | 0.827023368 | 0.113 |
| CD58 | NM_001779.2:478 | 0.880869374 | 0.469 |
| CD59 | NM_000611.4:730 | 3.555370725 | 0.00431 |
| CD6 | NM_006725.3:1280 | 0.153893052 | 0.0447 |
| CD7 | NM_006137.6:440 | 0.241484082 | 0.00598 |
| CD70 | NM_001252.2:190 | 19.15965927 | 0.0122 |
| CD74 | NM_001025159.1:964 | 2 | 0.0462 |
| CD79B | NM_021602.2:24 | 1.022570279 | 0.868 |
| CD80 | NM_005191.3:1288 | 6.36429187 | 0.00633 |
| CD81 | NM_004356.3:735 | 1.568080908 | 0.00127 |
| CD82 | NM_002231.3:1211 | 0.610050255 | 0.0961 |
| CD83 | NM_004233.3:1960 | 0.22221067 | 0.0373 |
| CD86 | NM_175862.3:1265 | 10.056107 | 0.0832 |
| CD8A | NM_001768.5:1320 | 1.181811547 | 0.31 |
| CD96 | NM_005816.4:1355 | 0.79940583 | 0.177 |
| CD97 | NM_078481.2:1370 | 0.411795509 | 0.00683 |
| CD99 | NM_002414.3:625 | 0.991098259 | 0.958 |
| CDKN1A | NM_000389.2:1975 | 2.329467173 | 0.00363 |
| CEBPB | NM_005194.2:1420 | 0.530343871 | 0.0342 |
| CFH | NM_001014975.2:702 | 4.789914818 | 0.00355 |
| CHUK | NM_001278.3:860 | 0.680657058 | 0.00263 |
| CISH | NM_145071.2:558 | 0.395020656 | 0.0114 |
| CMKLR1 | NM_004072.1:770 | 0.049036506 | 0.000784 |
| CRADD | NM_003805.3:675 | 0.720964436 | 0.0542 |
| CSF1 | NM_000757.4:823 | 2.099433367 | 0.0784 |
| CSF2 | NM_000758.2:475 | 0.463294031 | 0.242 |
| CSF2RB | NM_000395.2:3300 | 63.11889309 | 0.000589 |
| CTNNB1 | NM_001098210.1:1815 | 0.880869374 | 0.287 |
| CTSC | NM_001814.4:260 | 0.563700206 | 0.0518 |
| CTSS | NM_004079.3:685 | 0.906261938 | 0.2 |
| CUL9 | NM_015089.2:2675 | 0.623732786 | 0.00848 |
| CX3CR1 | NM_001337.3:1040 | 0.03794359 | 0.00845 |
| CXCR2 | NM_001557.2:2055 | 0.143587294 | 0.00937 |
| **CXCR3** | **NM_001504.1:80** | **2.188587403** | **0.082** |
| CXCR4 | NM_003467.2:1335 | 1.223488041 | 0.485 |
| **CXCR6** | **NM_006564.1:95** | **8.0556444** | **0.00502** |
| DPP4 | NM_001935.3:2700 | 2.928171392 | 0.122 |
| DUSP4 | NM_057158.2:3115 | 1.660940048 | 0.394 |
| EGR1 | NM_001964.2:1505 | 1.060908328 | 0.777 |
| EGR2 | NM_000399.3:1891 | 0.125 | 0.0835 |
| ENTPD1 | NM_001098175.1:8830 | 19.02731384 | 0.000874 |
| **EOMES** | **NM_005442.2:1670** | **1.736280455** | **0.0562** |
| ETS1 | NM_005238.3:4625 | 1.154285418 | 0.313 |
| FADD | NM_003824.2:1560 | 1.442928687 | 0.164 |
| FAS | NM_000043.3:90 | 0.813379198 | 0.332 |
| FCER1G | NM_004106.1:36 | 1.939923821 | 0.0412 |
| FCGR2A/C | NM_201563.4:1166 | 1.719512972 | 0.0171 |
| FCGR3A/B | NM_000570.4:696 | 1.625631204 | 0.159 |
| FKBP5 | NM_001145775.1:540 | 0.297301779 | 0.0295 |
| FYN | NM_002037.3:765 | 0.395020656 | 0.0194 |
| GATA3 | NM_001002295.1:2835 | 0.69640574 | 0.402 |
| GBP1 | NM_002053.1:2110 | 0.098755164 | 0.00289 |
| GBP5 | NM_052942.3:1955 | 0.41754396 | 0.0055 |
| GFI1 | NM_005263.2:2235 | 1.390881972 | 0.00508 |
| GNLY | NM_006433.2:305 | 3.294364069 | 0.0306 |
| GPI | NM_000175.2:1695 | 3.052518418 | 0.000312 |
| **GZMA** | **NM_006144.2:155** | **3.530811985** | **0.00453** |
| **GZMB** | **NM_004131.3:540** | **0.460093825** | **0.0214** |
| **GZMK** | **NM_002104.2:700** | **53.81737058** | **0.000204** |
| HAVCR2 | NM_032782.3:955 | 0.708578698 | 0.215 |
| HLA-A | NM_002116.5:1000 | 0.502083772 | 0.0427 |
| HLA-B | NM_005514.6:937 | 0.406126198 | 0.0152 |
| HLA-C | NM_002117.4:895 | 0.386891248 | 0.0168 |
| HLA-DMA | NM_006120.3:380 | 2.948538435 | 0.0158 |
| HLA-DMB | NM_002118.3:20 | 3.555370725 | 0.0554 |
| HLA-DOB | NM_002120.3:230 | 0.392292049 | 0.038 |
| HLA-DPA1 | NM_033554.2:857 | 4.0278222 | 0.0276 |
| HLA-DPB1 | NM_002121.4:931 | 2.566851795 | 0.145 |
| HLA-DQA1 | NM_002122.3:261 | 1.587767862 | 0.751 |
| HLA-DQB1 | NM_002123.3:384 | 3.630076621 | 0.29 |
| HLA-DRA | NM_019111.3:335 | 3.91768119 | 0.237 |
| HLA-DRB1 | NM_002124.2:104 | 3.944930818 | 0.164 |
| HLA-DRB3 | NM_022555.3:698 | 5.775716782 | 0.102 |
| HRAS | NM_005343.2:396 | 0.51370072 | 0.0115 |
| ICAM1 | NM_000201.2:2253 | 0.514056913 | 0.0789 |
| ICAM2 | NM_000873.3:415 | 0.23981603 | 0.0417 |
| ICAM3 | NM_002162.3:1225 | 1.247465572 | 0.00887 |
| ICOS | NM_012092.2:640 | 1.616641738 | 0.0355 |
| IFI16 | NM_005531.1:2255 | 0.927873476 | 0.0996 |
| IFI35 | NM_005533.3:415 | 0.420448208 | 0.00346 |
| IFIH1 | NM_022168.2:185 | 0.207329886 | 0.0308 |
| IFIT2 | NM_001547.4:1995 | 0.162667732 | 0.0257 |
| IFITM1 | NM_003641.3:482 | 0.882091365 | 0.0522 |
| IFNAR1 | NM_000629.2:3123 | 0.463294031 | 0.0703 |
| IFNAR2 | NM_000874.3:631 | 0.934651216 | 0.419 |
| **IFNG** | **NM_000619.2:970** | **1.890804234** | **0.168** |
| IFNGR1 | NM_000416.1:1140 | 0.231647015 | 0.00244 |
| IGF2R | NM_000876.1:2605 | 0.444421341 | 0.0594 |
| IKBKAP | NM_003640.3:1130 | 0.622437118 | 0.00251 |
| IKBKB | NM_001556.1:1995 | 0.469761375 | 0.0156 |
| IKBKE | NM_014002.2:2470 | 1.318593614 | 0.0152 |
| IKBKG | NM_003639.2:470 | 0.493116352 | 0.019 |
| IKZF1 | NM_006060.3:4485 | 0.741233505 | 0.103 |
| IKZF2 | NM_016260.2:870 | 0.813379198 | 0.274 |
| IKZF3 | NM_183232.2:1176 | 0.69399636 | 0.0463 |
| IL10RA | NM_001558.2:150 | 0.67877249 | 0.0327 |
| IL11RA | NM_147162.1:400 | 0.783497187 | 0.155 |
| IL12RB1 | NM_005535.1:225 | 0.435275282 | 0.0231 |
| IL15 | NM_172174.1:1685 | 1.003903084 | 0.972 |
| IL16 | NM_004513.4:1262 | 1.076240125 | 0.402 |
| IL18 | NM_001562.2:48 | 1.222640278 | 0.2 |
| IL18R1 | NM_003855.2:2025 | 0.482968164 | 0.105 |
| IL18RAP | NM_003853.2:2412 | 0.336808394 | 0.0295 |
| IL1RAP | NM_002182.2:460 | 1.468150636 | 0.00436 |
| IL1RL1 | NM_016232.4:700 | 3.482202253 | 0.0198 |
| IL21R | NM_021798.2:2080 | 0.378929142 | 0.107 |
| IL2RA | NM_000417.1:1000 | 0.604997045 | 0.252 |
| IL2RB | NM_000878.2:1980 | 1.922521857 | 0.0528 |
| IL2RG | NM_000206.1:595 | 0.803293997 | 0.326 |
| IL32 | NM_001012633.1:758 | 0.363493129 | 0.00442 |
| IL4R | NM_000418.2:705 | 0.596667872 | 0.0193 |
| IL6ST | NM_002184.2:2505 | 0.542238704 | 0.000209 |
| IL7R | NM_002185.2:1610 | 0.029769937 | 0.0462 |
| ILF3 | NM_001137673.1:730 | 0.701249625 | 0.0202 |
| IRAK1 | NM_001569.3:1995 | 0.592135806 | 0.0114 |
| IRAK2 | NM_001570.3:1285 | 0.752101876 | 0.232 |
| IRAK4 | NM_016123.1:2175 | 0.609205132 | 0.0556 |
| IRF1 | NM_002198.1:510 | 0.2381595 | 0.0146 |
| IRF3 | NM_001571.5:1303 | 0.782411782 | 0.0089 |
| IRF4 | NM_002460.1:325 | 0.496546248 | 0.191 |
| IRF5 | NM_002200.3:1845 | 1.748357241 | 0.00365 |
| IRF7 | NM_001572.3:1763 | 0.303548721 | 0.0286 |
| IRF8 | NM_002163.2:253 | 1.072516617 | 0.568 |
| ITGA4 | NM_000885.4:975 | 0.865736566 | 0.435 |
| ITGA5 | NM_002205.2:925 | 0.564873607 | 0.0081 |
| ITGA6 | NM_000210.1:3065 | 0.068393356 | 0.00303 |
| ITGAE | NM_002208.4:3405 | 0.473028823 | 0.00493 |
| ITGAL | NM_002209.2:3905 | 0.86154616 | 0.264 |
| ITGAM | NM_000632.3:515 | 0.559030925 | 0.0235 |
| ITGAX | NM_000887.3:700 | 2.828427125 | 0.00287 |
| ITGB1 | NM_033666.2:2000 | 0.47963206 | 0.00401 |
| ITGB2 | NM_000211.2:520 | 1.042971691 | 0.474 |
| JAK1 | NM_002227.1:285 | 0.400534939 | 0.00159 |
| JAK2 | NM_004972.2:455 | 0.571965487 | 0.055 |
| JAK3 | NM_000215.2:1715 | 0.665264521 | 0.00363 |
| **KIR_Activating_Subgroup_1** | **NM_001083539.1:1146** | **2.462288827** | **0.00828** |
| **KIR_Activating_Subgroup_2** | **NM_014512.1:718** | **3.073750363** | **0.00388** |
| **KIR_Inhibiting_Subgroup_1** | **NM_014218.2:872** | **3.052518418** | **0.00259** |
| **KIR_Inhibiting_Subgroup_2** | **NM_014511.3:592** | **3.052518418** | **0.00128** |
| **KIR3DL1** | **NM_013289.2:1691** | **1.325007017** | **0.306** |
| **KIR3DL2** | **NM_006737.2:884** | **1.396678532** | **0.366** |
| KIT | NM_000222.2:2644 | 0.40332088 | 0.0554 |
| KLRAP1 | NR_028045.1:414 | 1.994462503 | 0.234 |
| KLRB1 | NM_002258.2:85 | 1.437936533 | 0.377 |
| KLRC1 | NM_002259.3:335 | 1.643760375 | 0.143 |
| KLRC2 | NM_002260.3:942 | 1.742308384 | 0.094 |
| KLRC3 | NM_007333.2:598 | 0.958532996 | 0.634 |
| KLRC4 | NM_013431.2:276 | 0.724471077 | 0.0328 |
| KLRD1 | NM_002262.3:542 | 0.571965487 | 0.0258 |
| KLRF1 | NM_016523.1:275 | 0.548665969 | 0.113 |
| KLRF2 | NM_001190765.1:535 | 7.160200567 | 0.0217 |
| KLRG1 | NM_005810.3:65 | 0.721964598 | 0.301 |
| KLRK1 | NM_007360.1:760 | 1.068657834 | 0.636 |
| LAG3 | NM_002286.5:1735 | 5.464161027 | 0.0051 |
| LAIR1 | NM_002287.3:1195 | 0.824733549 | 0.11 |
| LCK | NM_005356.2:1260 | 1.054895434 | 0.604 |
| LCP2 | NM_005565.3:1210 | 0.695923196 | 0.115 |
| LEF1 | NM_016269.3:1165 | 0.332171454 | 0.0204 |
| LGALS3 | NM_001177388.1:495 | 0.432268616 | 0.194 |
| LIF | NM_002309.3:1240 | 0.609627547 | 0.254 |
| LILRA3 | NM_006865.3:1123 | 0.273573425 | 0.0144 |
| LILRB1 | NM_001081637.1:2332 | 1.266634254 | 0.519 |
| LITAF | NM_004862.3:1000 | 0.496546248 | 0.00833 |
| LTA | NM_000595.2:885 | 0.186856156 | 0.0575 |
| LTB4R | NM_181657.3:2400 | 1.093535457 | 0.49 |
| LTB4R2 | NM_019839.4:1250 | 0.880259014 | 0.2 |
| MALT1 | NM_006785.2:909 | 0.659753955 | 0.041 |
| MAP4K1 | NM_007181.3:780 | 1.096571589 | 0.166 |
| MAP4K2 | NM_004579.2:1610 | 0.85027416 | 0.0825 |
| MAP4K4 | NM_004834.3:3315 | 0.682073917 | 0.0641 |
| MAPK1 | NM_138957.2:430 | 0.671751713 | 0.00385 |
| MAPK14 | NM_001315.1:450 | 0.501388218 | 0.0054 |
| MAPKAPK2 | NM_004759.3:710 | 0.71400199 | 0.00855 |
| MBP | NM_002385.2:132 | 0.47963206 | 0.00583 |
| MCL1 | NM_021960.3:1260 | 0.942784536 | 0.345 |
| MIF | NM_002415.1:319 | 2.514026749 | 0.0047 |
| MR1 | NM_001531.2:7695 | 0.583174685 | 0.0081 |
| MX1 | NM_002462.2:1485 | 0.356012549 | 0.135 |
| MYD88 | NM_002468.3:2145 | 0.60583633 | 0.0267 |
| NCAM1 | NM_000615.5:1620 | 2.549121255 | 0.00973 |
| NCF4 | NM_000631.4:210 | 35.26096371 | 0.00236 |
| NCR1 | NM_004829.5:602 | 0.868742185 | 0.367 |
| NFATC1 | NM_172389.1:1984 | 0.635075491 | 0.0707 |
| NFATC2 | NM_012340.3:1815 | 0.231647015 | 0.0117 |
| NFATC3 | NM_004555.2:2190 | 0.980575078 | 0.867 |
| NFIL3 | NM_005384.2:1795 | 2.313376368 | 0.00757 |
| NFKB1 | NM_003998.2:1675 | 0.406126198 | 0.0116 |
| NFKB2 | NM_002502.2:825 | 0.214641359 | 0.00184 |
| NFKBIA | NM_020529.1:945 | 0.411795509 | 0.0111 |
| NFKBIZ | NM_001005474.1:2030 | 0.664342907 | 0.179 |
| NOD1 | NM_006092.1:3285 | 0.920825697 | 0.312 |
| NOD2 | NM_022162.1:4080 | 3.732131966 | 0.00837 |
| NOTCH1 | NM_017617.3:735 | 0.476318999 | 0.0186 |
| NOTCH2 | NM_024408.3:2842 | 0.638164384 | 0.0258 |
| PDCD2 | NM_144781.2:288 | 0.603740296 | 0.0194 |
| PDGFRB | NM_002609.3:840 | 0.522317881 | 0.334 |
| PECAM1 | NM_000442.3:1365 | 2.887858391 | 0.00544 |
| PLAU | NM_002658.2:793 | 0.194791145 | 0.0987 |
| PML | NM_002675.3:281 | 0.600818025 | 0.112 |
| POU2F2 | NM_002698.2:908 | 0.129408115 | 0.0139 |
| PRDM1 | NM_001198.3:798 | 0.828170661 | 0.252 |
| PRF1 | NM_005041.3:2120 | 0.932386486 | 0.57 |
| PRKCD | NM_006254.3:2165 | 0.469761375 | 0.000705 |
| PSMB10 | NM_002801.2:221 | 0.543744195 | 0.00529 |
| PSMB5 | NM_001130725.1:716 | 0.693515485 | 0.0307 |
| PSMB7 | NM_002799.2:420 | 0.839731493 | 0.0535 |
| PSMB8 | NM_004159.4:1215 | 0.687770909 | 0.00588 |
| PSMB9 | NM_002800.4:455 | 0.650220073 | 0.0117 |
| PSMC2 | NM_002803.3:446 | 0.744838732 | 0.0423 |
| PSMD7 | NM_002811.3:580 | 0.732042848 | 0.0566 |
| PTGER4 | NM_000958.2:1380 | 0.688725023 | 0.122 |
| PTPN2 | NM_002828.2:185 | 0.373712312 | 0.0053 |
| PTPN22 | NM_015967.4:2505 | 1.4054187 | 0.0736 |
| PTPN6 | NM_002831.5:1734 | 1.349102534 | 0.00359 |
| PTPRC_all | NM_080921.2:90 | 0.625898229 | 0.0637 |
| PYCARD | NM_013258.3:714 | 1.797510253 | 0.0865 |
| RAF1 | NM_002880.2:1990 | 0.646176415 | 0.0351 |
| RARRES3 | NM_004585.3:640 | 0.854409741 | 0.493 |
| RELA | NM_021975.2:360 | 0.406126198 | 0.00124 |
| RELB | NM_006509.2:250 | 0.176776695 | 0.00788 |
| RUNX1 | NM_001754.4:635 | 1.070882357 | 0.566 |
| S1PR1 | NM_001400.3:1065 | 0.115823508 | 0.00958 |
| sCTLA4 | NM_001037631.1:560 | 1.278985581 | 0.375 |
| SELL | NR_029467.1:1585 | 0.055552668 | 0.0185 |
| SELPLG | NM_003006.3:2297 | 0.339151082 | 0.0216 |
| SH2D1A | NM_001114937.2:495 | 3.555370725 | 0.00246 |
| SIGIRR | NM_021805.2:469 | 0.489710149 | 0.0654 |
| SKI | NM_003036.2:3335 | 0.295248165 | 8.07E-05 |
| SLAMF6 | NM_001184714.1:1032 | 0.85797053 | 0.363 |
| SLAMF7 | NM_021181.3:215 | 0.808881348 | 0.359 |
| SLC2A1 | NM_006516.2:2500 | 1.472226862 | 0.0694 |
| SMAD3 | NM_005902.3:4220 | 0.489710149 | 0.0198 |
| SMAD5 | NM_005903.5:1044 | 0.574747424 | 0.00172 |
| SOCS1 | NM_003745.1:1025 | 0.26425451 | 0.000971 |
| SOCS3 | NM_003955.3:1870 | 0.524858342 | 0.0798 |
| SRC | NM_005417.3:1410 | 1.474269217 | 0.0479 |
| STAT1 | NM_007315.2:205 | 0.509798841 | 0.0424 |
| STAT2 | NM_005419.2:1965 | 0.544876056 | 0.0171 |
| STAT3 | NM_139276.2:4535 | 0.504875649 | 0.0487 |
| STAT4 | NM_003151.2:789 | 0.628942486 | 0.0382 |
| STAT5A | NM_003152.2:3460 | 0.631563631 | 0.0325 |
| STAT5B | NM_012448.3:200 | 0.642157904 | 0.0157 |
| STAT6 | NM_003153.3:2030 | 0.408951029 | 0.0194 |
| SYK | NM_003177.3:1685 | 1.308578071 | 0.277 |
| TAGAP | NM_054114.3:169 | 0.564482202 | 0.0291 |
| TAP1 | NM_000593.5:2075 | 0.348685917 | 0.00553 |
| TAP2 | NM_000544.3:909 | 0.329876978 | 0.00294 |
| TAPBP | NM_003190.4:1536 | 0.532554102 | 0.0371 |
| TBK1 | NM_013254.2:1610 | 0.890075733 | 0.0312 |
| **TBX21** | **NM_013351.1:890** | **0.636838738** | **0.0073** |
| TCF4 | NM_003199.1:1455 | 1.674812975 | 0.111 |
| TCF7 | NM_003202.2:2420 | 0.05440941 | 0.00171 |
| TFRC | NM_003234.1:1220 | 1.048843917 | 0.434 |
| TGFB1 | NM_000660.3:1260 | 0.489710149 | 0.00577 |
| TGFBR1 | NM_004612.2:4280 | 0.548665969 | 0.123 |
| TGFBR2 | NM_001024847.1:1760 | 0.849684999 | 0.425 |
| TICAM1 | NM_014261.1:518 | 0.544121221 | 0.0326 |
| TIGIT | NM_173799.2:1968 | 0.543367431 | 0.104 |
| TIRAP | NM_148910.2:661 | 0.61429349 | 0.0567 |
| TLR1 | NM_003263.3:545 | 0.969491396 | 0.881 |
| TMEM173 | NM_198282.1:725 | 1.798756624 | 0.13 |
| TNF | NM_000594.2:1010 | 0.892546971 | 0.687 |
| TNFAIP3 | NM_006290.2:260 | 0.241484082 | 0.0423 |
| TNFRSF13C | NM_052945.3:789 | 8.224910613 | 0.132 |
| TNFRSF14 | NM_003820.2:916 | 0.473028823 | 0.0101 |
| TNFRSF1B | NM_001066.2:835 | 0.563700206 | 0.0926 |
| TNFRSF4 | NM_003327.2:200 | 0.368567304 | 0.133 |
| TNFRSF9 | NM_001561.4:255 | 0.10438599 | 0.0168 |
| TNFSF10 | NM_003810.2:115 | 0.821310701 | 0.1 |
| TNFSF11 | NM_003701.2:490 | 1.043477867 | 0.835 |
| TNFSF12 | NM_003809.2:339 | 0.991854221 | 0.862 |
| TNFSF13B | NM_006573.4:1430 | 1.223488041 | 0.203 |
| TNFSF4 | NM_003326.2:545 | 0.602486157 | 0.305 |
| TNFSF8 | NM_001244.3:518 | 1.792533399 | 0.171 |
| TOLLIP | NM_019009.2:1320 | 0.72597914 | 0.0245 |
| TP53 | NM_000546.2:1330 | 0.629378587 | 0.023 |
| TRAF1 | NM_005658.3:3735 | 0.204475515 | 0.0268 |
| TRAF2 | NM_021138.3:1325 | 0.62676651 | 0.0172 |
| TRAF3 | NM_145725.1:1795 | 0.660669203 | 0.0076 |
| TRAF4 | NM_004295.2:1060 | 0.819604608 | 0.0382 |
| TRAF5 | NM_004619.3:400 | 1.155886707 | 0.0746 |
| TRAF6 | NM_145803.1:1839 | 0.557096825 | 0.0263 |
| TYK2 | NM_003331.3:485 | 0.500693628 | 0.00676 |
| UBE2L3 | NM_198157.1:990 | 0.698339266 | 0.0169 |
| XBP1 | NM_005080.2:440 | 0.586824089 | 0.0233 |
| XCL1 | NM_002995.1:620 | 0.069348092 | 0.00401 |
| ZAP70 | NM_001079.3:1175 | 0.47963206 | 0.00488 |
| ZBTB16 | NM_006006.4:1585 | 0.748461493 | 0.119 |
| ZEB1 | NM_001128128.1:1450 | 2.313376368 | 0.0113 |
