# Supplementary material for: An Uncoupling of Canonical Phenotypic Markers and Functional Potency of Ex Vivo-Expanded Natural Killer Cells
Source: Front Immunol. 2018 Feb 2;9:150. doi: 10.3389/fimmu.2018.00150 (PMC5801405; doi:10.3389/fimmu.2018.00150)
Supplement: Supplementary file 3 [file Image_1.PDF]

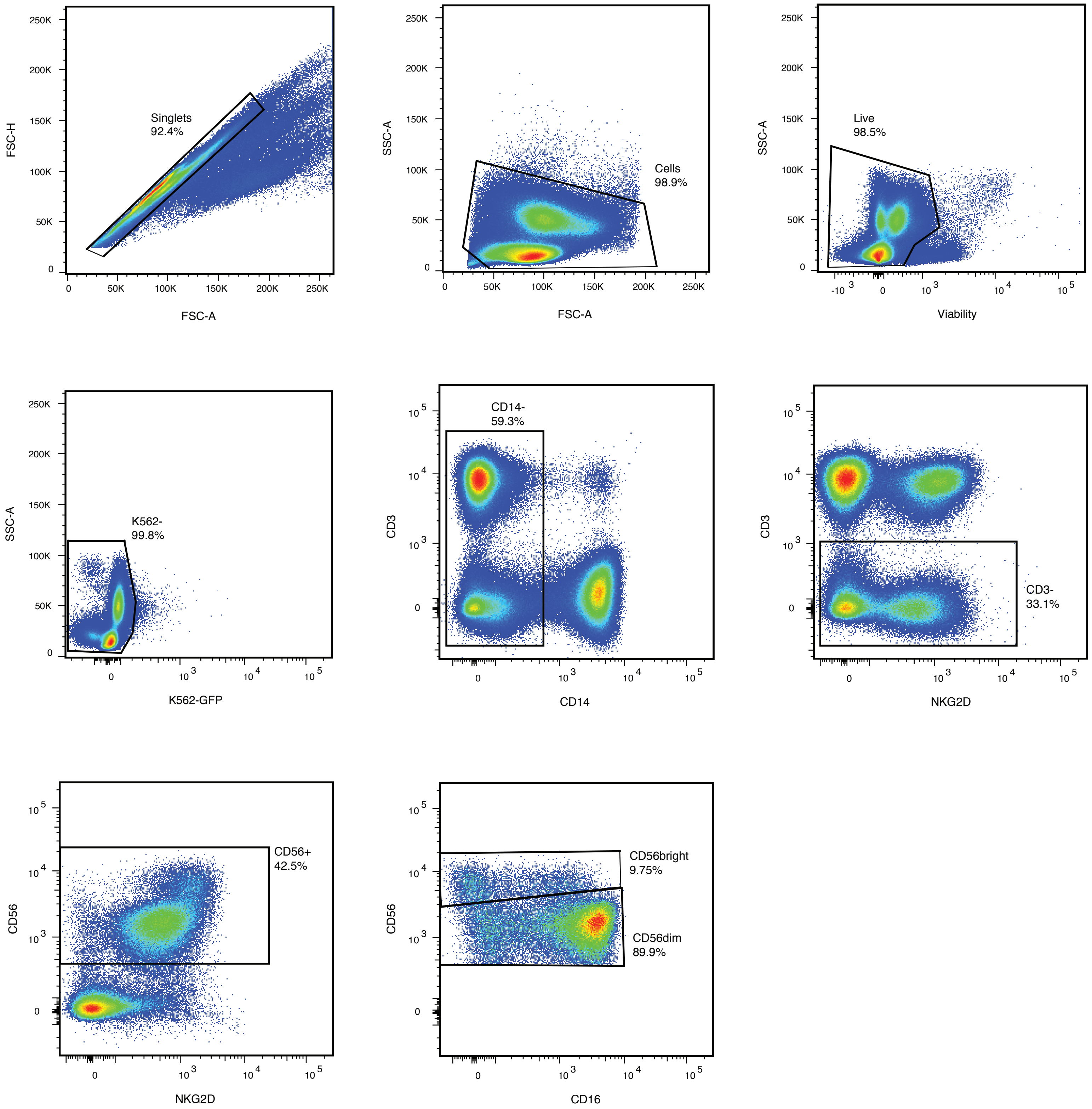

Supplemental Figure 1: Gating strategy for NK cells. Singlets, debris, dead cells, and residual K562 cells, which express GFP, are removed. Monocytes expressing CD14 are excluded, followed by all CD3+ cells. All CD56+ cells are gated, and subset analysis performed by CD16 expression.
