# Supplementary material for: An Uncoupling of Canonical Phenotypic Markers and Functional Potency of Ex Vivo-Expanded Natural Killer Cells
Source: Front Immunol. 2018 Feb 2;9:150. doi: 10.3389/fimmu.2018.00150 (PMC5801405; doi:10.3389/fimmu.2018.00150)
Supplement: Supplementary file 4 [file Image_2.PDF]

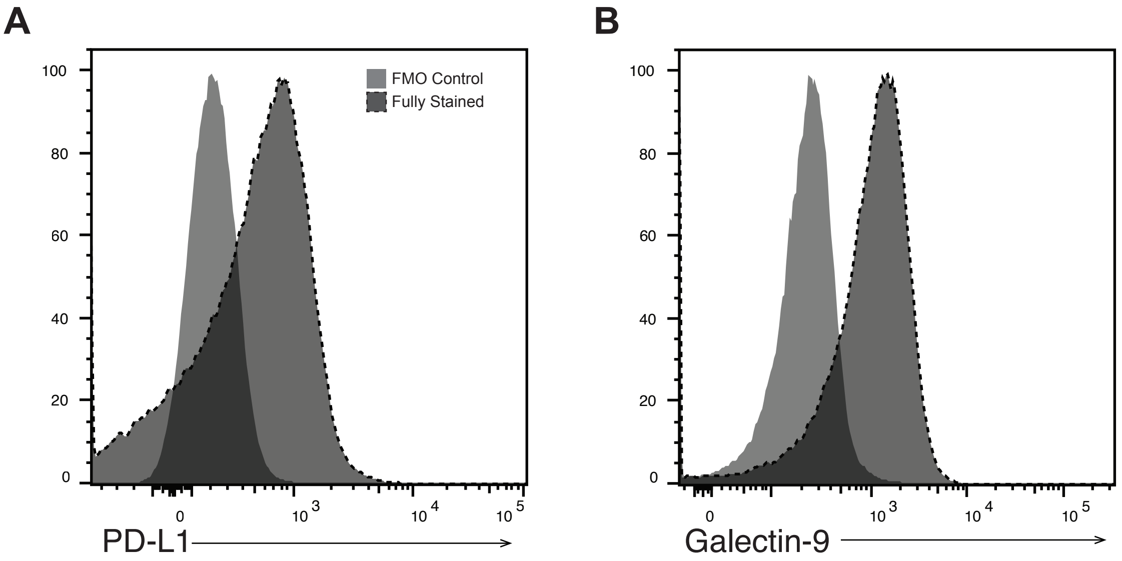

Supplementary Figure 2: Exhaustion ligand expression on K562. Fluorescence minus one (FMO) control (solid light grey) and fully stained (dashed line, dark grey) K562 samples for PD-L1 (A) and Gal-9 (B).
